# The Scenario of Ticks and Tick-Borne Pathogens of Sheep on a Mediterranean Island

**DOI:** 10.3390/microorganisms10081551

**Published:** 2022-07-31

**Authors:** Anastasios Saratsis, Panagiota Ligda, Fredie Aal, Mandy Jelicic, Juliette Polgar, Myrthe de Vries, Ioannis Mastranestasis, Vincenzo Musella, Laura Rinaldi, Frans Jongejan, Smaragda Sotiraki

**Affiliations:** 1 Veterinary Research Institute, Hellenic Agricultural Organisation-Demeter, 57001 Thessaloniki, Greece; giota.lig@hotmail.com (P.L.); mastranestasis@yahoo.gr (I.M.); 2 Utrecht Centre for Tick-Borne Diseases (UCTD), FAO Reference Centre for Ticks and Tick-Borne Diseases, Faculty of Veterinary Medicine, Utrecht University, Yalelaan 1, 3584 CL Utrecht, The Netherlands; fredie.aal@hotmail.com (F.A.); mandy_jelicic@hotmail.com (M.J.); j.a.polgar@uu.nl (J.P.); myrthedv@gmail.com (M.d.V.); or FJongejan@TBDInternationalBV.com (F.J.); 3 Department of Health Science, University “Magna Græcia” of Catanzaro, Viale Europa, 88100 Catanzaro, Italy; musella@unicz.it; 4 Department of Veterinary Medicine and Animal Production, University of Naples Federico II, CREMOPAR Campania Region, Via Della Veterinaria 1, 80137 Naples, Italy; lrinaldi@unina.it; 5 TBD International B.V., Ramstraat 39, 3581 HE Utrecht, The Netherlands; 6 Department of Veterinary Tropical Diseases, Faculty of Veterinary Science, University of Pretoria, Private Bag X04, Onderstepoort, Pretoria 0110, South Africa

**Keywords:** *Rhipicephalus turanicus*, *Anaplasma ovis*, Ixodidae, tick-borne pathogens, sheep, PCR–reverse line blot

## Abstract

Ticks and transmitted pathogens constitute a major concern for livestock health/welfare and productivity for the Mediterranean region, often posing an important zoonotic threat. The aim of this study was to investigate the presence, infection intensity, and seasonality of ticks and tick-borne pathogens on the island of Lesvos in Greece, which was selected as a potential hotspot for their circulation. To this end, 101 sheep farms were visited over a tick activity season, and ticks, blood samples, and questionnaire data were collected. Ticks were identified by species, and DNA from both ticks and blood samples was further investigated using the polymerase chain reaction–reverse line blot (PCR–RLB) technique. In 72.3% of the farms, sheep were found to be infected by 9 ixodid species, with *Rhipicephalus turanicus* being the most common during the spring/early summer period. As regards tick-borne pathogens (TBPs), 84.9% of the animals were found to be infected with at least one pathogen, the most common being genera of *Anaplasma* and *Theileria*, alone or in co-infections. To further characterize the *Anaplasma* species found, selected samples were sequenced, revealing isolates of *A. ovis*, *A. capra*, *A. marginale*, and *A. phagocytophilum*. Of the 169 female *R. turanicus* ticks analyzed by PCR–RLB, 89.9% were harboring at least one TBP belonging to the genera *Anaplasma*, *Ehrlichia*, *Babesia*, *Theileria*, or *Rickettsia*. Overall, the data presented in this study revealed a high burden of ticks and TBPs in sheep, including zoonotic species, stressing the need for applying effective monitoring and control programs using a more holistic One Health approach.

## 1. Introduction

Ticks constitute a major concern for livestock health/welfare and productivity. This is due not only to their hematophagous behavior, but largely to their ability to transmit diseases [1]. The greater importance of ticks parasitizing livestock in Mediterranean countries compared to Northern European countries is documented by the higher number of published data on both ticks and tick-borne pathogens (TBPs) in the specific region [2]. In times of climate change, the impact on both animal and human health is becoming more obvious owing to the increasing number of alarming reports on both arthropod vector distributional/behavioral changes and their associated infectious agents [3]. For instance, a recent report correlated a cluster of Mediterranean spotted fever cases in humans with a warming-mediated increase in the aggressiveness of *Rhipicephalus sanguineus* sensu lato ticks [4]. In addition, ecological niche factor analysis and principal component analysis of climate variables predict an expansion of suitable habitats for common Mediterranean tick species such as *R. turanicus* and *Hyalomma marginatum* [5]. 

Tick-borne pathogens can cause significant losses in livestock reared outdoors in extensive systems, such as sheep [6]. Specifically, for the Mediterranean region, there are several surveys reporting high endemicity of the pathogens of the genus *Anaplasma* spp.—such as *A. phagocytophilum, A. ovis*, and *A. capra*—in sheep; however, their pathogenicity is not fully described [2,7,8,9,10,11,12]. In Greece, sheep farming is particularly important (with a national flock of 8,427,196 animals—Hellenic Statistical Authority, https://www.statistics.gr (accessed on 1 June 2022)), and the large majority of animals are dairy sheep grazing all year round and, hence, constantly exposed to tick infestations. Several studies addressing tick presence and distribution were conducted in the past in Greece, revealing the presence of over 20 hard tick (*Ixodidae*) species parasitizing domestic and wild animals [13,14,15,16,17,18,19,20,21]. However, serological and/or molecular epidemiological data related to TBPs in small ruminants are scarce and/or outdated and refer to specific pathogens [22,23,24,25,26]. The latter is becoming more important, since there is evidence that on several occasions pathogens occur simultaneously in both the host and the tick vector, and this has been observed more frequently than previously thought [27,28].

The aims of this study were to investigate the presence, abundance, and seasonal distribution of hard tick species infesting sheep, molecularly identify the TBPs present in both sheep and ticks in order to identify host–vector–pathogen dependence, and determine the epidemiological importance of exposure of sheep to TBPs. 

## 2. Materials and Methods

### 2.1. Study Area and Sheep Farming

To address the above objectives, the present study was performed in a geographically restricted location with a high risk of tick infestations (i.e., an island with high sheep farm density and suitable climatic conditions), and included repeated samplings of sheep over a tick activity season. 

Specifically, the study was performed on the island of Lesvos, Greece, which is the third largest island in the country, covering a surface of 1636 m^2^. It is located in the northeastern part of the Aegean Sea, and constitutes a regional unit of the North Aegean Region. With an annual mean temperature of 18 °C and a mean annual rainfall of 750 mm, the climate is typical mild Mediterranean, with different habitat types consisting of shrubs, olive tree orchards, and pine forests [29]. Moreover, the island of Lesvos is situated along the western Black Sea bird migration route, being considered an important migration stopover site [30]. Sheep farming on the island of Lesvos has a longstanding tradition, with a recorded population of 376,811 sheep bred under semi-intensive dairy production systems, with the majority of them belonging to the local Lesvos breed [31,32,33]. 

### 2.2. Study Design

To better design the sampling procedure, data on the number of farms on the island and their exact coordinates were retrieved from the national registry (North Aegean Region, Directorate of Rural Economy and Veterinary Services). The estimation of animals to be sampled was performed according to https://www.openepi.com (accessed on 5 February 2019), using the following values in the equation for cross-sectional studies: 376,000 as the total number of animals, 50% prevalence, 97% confidence interval, 5% desired absolute precision, and 1 for design effect. This resulted in 471 animals to be sampled, and was achieved by means of a grid approach followed by proportional allocation. More precisely, a grid consisting of 5 × 5 km quadrants was laid over the island, and the total number of farms in each of them was defined. Subsequently, the sample size was proportionally allocated (by considering systematic sampling from 5 adult animals per farm) into the respective quadrants, and farms were randomly selected (the number of animals to be sampled in each quadrant was proportional to the total number of farms in each of them). The above procedure was performed by using GIS software (GIS, ArcGIS version 10.2.2, ESRI, Redlands, CA, USA). Sampling was organized based on two sampling periods: one in spring/early summer, and another in autumn. Specifically, samplings were performed in May, June, September, and October. The above periods were chosen based on farmers’ feedback about increased tick activity. Each sampling visit lasted a maximum of one week to avoid climatic deviations. Special care was taken to cover all selected quadrants in each visit. Farms were enrolled in the survey provided that animals were not treated with an acaricide for at least a month before the visit. 

Overall, 101 farms in total were visited during the study period; 18 farms were sampled in May, 21 farms in June, 32 farms in September, and 30 farms in October.

### 2.3. Questionnaire

A structured questionnaire was designed to obtain information on farm characteristics (i.e., farm location, land use, flock size and structure, facilities and equipment, presence of other animals), common health problems—including awareness of farmers with regard to TBP-related health issues (i.e., recording of general health problems, observations on tick activity throughout the year, signs of fever, recumbency, appetite loss, hemoglobinuria, swollen lymph nodes, paralysis, and death related to tick presence)—and applied management practices, with particular relevance to ticks and TBPs (i.e., pasture size availability, grazing practices and duration for every month of the year, drugs used, their costs, and frequency of treatments against ticks and TBPs per year). The above data were subsequently statistically evaluated as potential risk factors for the presence of the most common pathogens detected during the survey. 

### 2.4. Blood Sampling and Tick Collection

Selected animals per farm were thoroughly checked for the presence of ticks at the head (ears, horns), udder, perianal region, hindlegs, tail, and withers/back. Subsequently, a blood sample was collected from each animal in an EDTA tube, maintained in a cool box at 4 °C, and stored at −20 °C until further analyses. Ticks were placed in numbered and dated tubes containing 70% ethanol immediately after collection, and identified according to specific keys [34,35].

### 2.5. DNA Extraction from Blood and Ticks

DNA was extracted from the collected blood samples (*n* = 505) using the DNeasy^®^ Blood & Tissue kit (Qiagen, Hilden, Germany), according to the manufacturer’s instructions. One hundred and sixty-nine engorged, adult, female *R. turanicus* ticks were selected for DNA isolation, using the NucleoSpin Tissue Kit (Macherey-Nagel, Düren, Germany), as previously described [36]. Every single tick was collected from a different animal representing all animals infested with at least one tick of this species. DNA samples from blood and tick specimens were stored at −20 °C until further analyses. 

### 2.6. PCR Amplification

PCR amplification and a reverse line blot hybridization (RLB) assay were employed for both blood and tick sample analysis. For this, standard RLB membranes used at the UCTD for TBP detection were used. These membranes were designed to simultaneously detect all common species of the genera *Ehrlichia*, *Neoehrlichia*, *Anaplasma*, *Theileria*, *Babesia*, and *Rickettsia*. *Ehrlichia*/*Anaplasma* PCR was performed using the primers Ehr-F (5′-GGAATTCAGAGTTGGATCMTGGYTCAG-3′) and Ehr-R (5′-biotin-CGGGATCCC GAGTTTGCCGGGACTTYTTCT-3′), amplifying a fragment of the V1 hypervariable region of the 16S rRNA gene [37]. *Babesia/Theileria* PCR was performed using the primers RLB-F2 (5′-GACACAGGGAGGTAGTGACAAG-3′) and RLB-R2 (biotin-5′-CTAAGAATTTCACCTCTGACAGT-3′) to amplify a fragment of the 18S rRNA gene spanning the V4 region [38]. PCR reactions were performed as previously described [36]. Positive (i.e., DNA material confirmed by sequencing, derived from known clinical cases, kept as standards at the UCTD) and negative (i.e., PCR water) controls were included in each run, and the obtained PCR products were stored at −20 °C until further analyses.

### 2.7. Reverse Line Blot Hybridization (RLB) Assay

The RLB assay was performed as previously described [39]. Oligonucleotide probes containing an *N*-terminal *N*-(trifluoracetamidohexyl-cyanoethyl, *N*, *N*-diisopropyl phosphoramidite)-C6 amino linker (Eurogentec, Maastricht, the Netherlands) were covalently linked to the RLB membrane (Biodyne C blotting membrane; Pall Biosupport, Ann Arbor, MI, USA) using the following procedure: The membrane was activated by a 10 min incubation at room temperature in a freshly prepared 10 mL solution of 16% 1-ethyl 3-(3-dimethylaminopropyl) carbodiimide HCl (Sigma, St. Louis, MO, USA). The membrane was briefly rinsed in distilled water and then placed in a MN45 MiniBlotter (Immunetics, Cambridge, MA, USA), and the residue liquid was aspirated. The oligonucleotide probes were diluted to a concentration of 400 pmol/150 μL in 500 mM NaHCO_3_ solution (pH 8.4), and linked to the membrane by loading onto the lanes of the MiniBlotter. This was followed by a 1 min incubation, after which the probes were aspirated. After aspiration of the oligonucleotide probe solutions, the membrane was washed in a 100 mL freshly prepared 100 mM NaOH solution for 8 min at room temperature under gentle shaking to inactivate the membrane.

After inactivation, the membrane was washed for 5 min at 60 °C in a 2× SSPE (2× SSPE is 0.36 M NaCl, 20 mM NaH_2_PO_4_, and 2 mM EDTA (pH 7.7))−0.1% sodium dodecyl sulfate (SDS) solution under gentle shaking, followed by a washing step using a 20 mM EDTA solution for 15 min at room temperature under gentle shaking to rinse the membrane of any residue before storage in fresh 20 mM EDTA solution at 4 °C until further use. The RLB membrane was hybridized with the PCR products and further developed as previously described [40].

### 2.8. Sequencing

PCR products of representative samples found to be positive for *Anaplasma ovis* (*n* = 12) and all *Ehrlichia/Anaplasma* catch-all (*n* = 10) were sent to a commercial service (CeMIA SA, Larissa, Greece) for purification and sequencing on both strands (Sanger sequencing). The results were assembled with SeqMan 8.1 software (Lasergene DNASTAR, Madison, WI, USA). Assembled sequences were aligned using the Basic Local Alignment Search Tool (BLAST) and compared with reference sequences using MegAlign (Lasergene DNASTAR).

### 2.9. Statistics

Descriptive analysis (i.e., average values, standard deviations, and exposure rates), chi-squared tests (i.e., comparison of exposure rates between periods of sampling), and binary logistic regression analysis were performed using IBM SPSS statistics (version 23). The effects of independent variables (i.e., presence/absence and number of each tick species based on sheep gender, breed, pasture surface availability, period of sample collection, frequency of acaricide treatments per year, number of personnel, number of sheep per flock, and altitude) on the sheep’s exposure status (dependent dichotomous variable) to the two most common TBPs encountered during the study—namely, *A. ovis* and *T. ovis*—were analyzed through a binary logistic regression model with forward LR selection. Initially, a test of the full model against a constant-only model was performed in order to assess whether there was a statistically significant effect of the examined independent predictors on the response variable through the utilization of the Omnibus Tests of Model Coefficients, which use the chi-squared test to see if there is a significant difference between the log-likelihood (-2LL) of the baseline model (constant model) and that of the model with the predictors. In addition, the Hosmer–Lemeshow (H–L) test was performed to assess whether the model provided a good fit to the data. Model characteristics are provided in the Supplementary Materials (Table S1).

### 2.10. Ethical Considerations

This study was carried out in compliance with the national animal welfare regulations. Diagnostic veterinary procedures are not within the context of relevant EU legislation for animal experimentations (Directive 86/609/EC), and may be performed to diagnose animal diseases and improve animal welfare. To justify this, we obtained a statement of exclusion by the State Veterinary Services (ref number: 661526(2804) 1 December 2020).

Samples were collected by registered veterinarians and caused no suffering. All farm owners gave their informed consent during the interview for the purpose of the study. This study was part of a postdoctoral research project (ΚΥPΕ 7718/Β35) funded by national funds, and during evaluation it was also approved by an ethical committee.

## 3. Results

### 3.1. Farm Description (Questionnaire Data)

All farms were classified as semi-intensive, based on the fact that supplementary feeding with concentrates was provided in all of them throughout the year [41]. Of the flocks included in the survey, 88.4% had purebred animals belonging to the local Lesvos breed. The rest of them consisted mainly of either purebred high-yielding foreign and local dairy sheep breeds such as Lacaune, Aschaf, Friesian, and Chios, or crossbred animals of the local breed with the former breeds. Flocks had an average number of 257.5 ± 147.0 ewes (estimated mean weight of 51.5 ± 4.7 kg)—85.6% of which were milked during the survey period—63.3 ± 40.3 replacement lambs, and 11.6 ± 6.9 rams.

Grazing was applied in 100% of the flocks, with 24.1% of them reporting sharing of pastures with other flocks. In addition, owned and rented pastures were available for 96.2% (average pasture size 22.8 ± 23.7 hectares) and 89.9% (average pasture size 39.6 ± 35.8 hectares) of the flocks, respectively. It should be mentioned that the vast majority of the pastures were non-irrigated, with natural vegetation, whereas a considerable proportion were found within olive tree orchards. A total of 20.5% of the farmers also utilized communal pastures (average size of 30.8 ± 46.8 hectares). 

Tick infestation, irrespective of the month, was reported by 95.3% of the farmers. Depending on the month of the year, except for January and February, farm infestation rates with ticks were reported ranging between 5.9% and 90.6%. Peaks in tick activity were mainly observed in May and October, as stated by 90.6% and 57.6% of the farmers, respectively. Less than 10% (range 0–10%) of the farmers could establish a connection between the presence of ticks and typical tick-borne-infection-related clinical signs such as fever, appetite loss, hemoglobinuria, breathing problems, swollen lymph nodes, paralysis, or death. Only two farmers reported that they had to treat animals of their flocks for piroplasmosis (using imidocarb dipropionate), with treatment results considered satisfactory. Regarding ectoparasitic treatments, 96.4% of the farmers responded that they routinely use ectoparasiticides. Of those, 49.4%, 30.1%, and 20.5% routinely applied one, two, and three treatments per year, respectively. The most common acaricide classes used, either alone or in combination, were synthetic pyrethroids (80.5% of the farmers applied cypermethrin and 6.5% deltamethrin) and macrocyclic lactones (6.8% reported using either ivermectin or moxidectin). The average cost related to acaricide application per year was highly variable (EUR 171.4 to 298.4).

### 3.2. Tick Collection

**Figure 1:**
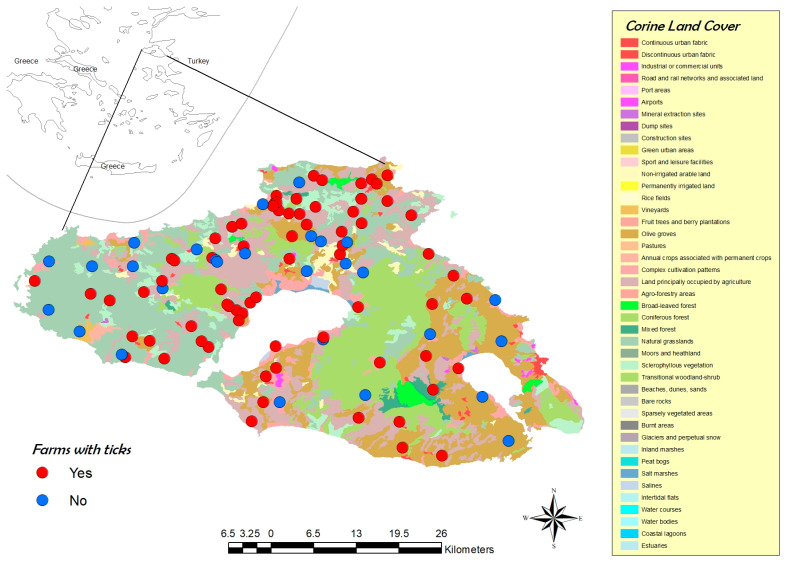
Distribution of sampled farms based on tick presence/absence and landscape fragmentation, according to CORINE land cover.

**Figure 2:**
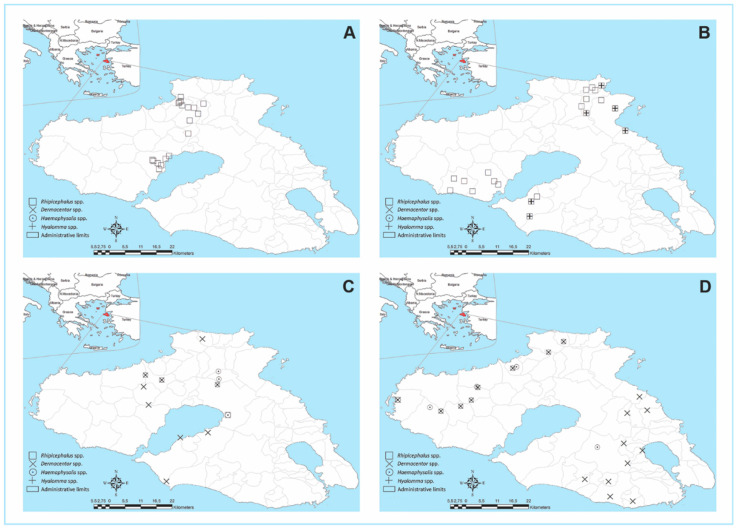
Tick genera found based on the month ((**A**) = May, (**B**) = June, (**C**) = September, (**D**) = October) of sampling and farm location.

In Figure 1 and Figure 2, the distribution of the 101 enrolled farms on the island, based on tick infestation/land-cover fragmentation and the period or month of sampling, can be seen. In 72.3% of the farms, tick infestation with nine different species was observed throughout the collection period (Table 1). Of the examined animals (*n* = 505), 55.4% were infested with ticks. Infestation rates at the animal level were higher during May–June (92.8%, with a mean infestation intensity of 6.41 ticks per animal and a range of 1–40) compared to the September-October collection period (31.9%, with an average infestation intensity of 5.58 ticks per animal and a range of 1–54). In total, 1707 adult ticks were collected (54% males and 46% females). No immature ticks were found.

During the spring/early summer sampling period, *R. turanicus* was the predominant species found, accounting for 95.2% and 84.5% of the collected ticks during May and June, respectively (Table 1). This species was found in 38 out of 39 farms, parasitizing 86.7% of sheep checked during this period. *Rhipicephalus sanguineus* s.l. (2.2% of collected ticks during this period), *R. bursa* (5.1%), *Hyalomma marginatum* (1.8%), *and H. excavatum* (0.7%) were also collected in small numbers during the spring/early summer period. The above species were collected from 10, 15, 5, and 3 out of 39 farms, respectively. During autumn (September–October), *Dermacentor marginatus* (48.8% of collected ticks during this period) and *Haemaphysalis parva* (43.7%) predominated. They were found in 27 and 16 out of 62 farms visited during the above period, respectively. In addition, *H. sulcata* (0.2%), *H. punctata* (7.0%), and *R. bursa* (0.4%) adults were collected in small numbers. The most common predilection sites were the ears (*Rhipicephalus* spp.), udder/tail/perianal region/axilla (*Hyalomma* and *Rhipicephalus* spp.), and withers (*D. marginatus* and *Haemaphysalis* spp.). *D. marginatus* ticks typically fed in batches, sometimes together with *Haemaphysalis* spp., while in some cases *Haemaphysalis* spp. were also collected from the ears. 

### 3.3. Prevalence of Tick-Borne Pathogens in Sheep

PCR–RLB was used to assess the exposure rates to TBPs in 505 sheep blood samples collected over the study period. In Table 2 and Table 3, the rates of single pathogen presence (multiple presence not considered), stratified based on the collection period or the prevalence of single combined with multiple presence, respectively, are presented (confidence intervals are provided in the respective tables). In total, 84.9% of the animals were found to be infected with at least one pathogen. Multiple presence with either two or three TBPs (Table 3) was observed in 57.4% and 0.8% of the samples, respectively. Sheep were found to be infected with up to four different TBPs (*A. ovis*, *Theileria ovis, Babesia ovis*, *A. bovis*), whereas in 9 (1.8%) and 10 cases (2.0%) the presence of one or more unknown *Babesia/Theileria* spp. and *Anaplasma/Ehrlichia* spp., respectively, was observed. The presence of multiple infections was mainly observed between *A. ovis* and *T. ovis* (53.4%). Exposure to *Rickettsia* spp. was not recorded in any of the animals.

The most prevalent TBP was *A. ovis*, with an overall prevalence of 77.2%. The exposure rate to *A. ovis* during the spring/early summer collection period (93.8%) was statistically significantly different (*p* < 0.001) compared to the autumn exposure rate (66.7%). This was not observed for the rest of the TBPs when the two periods were compared (Table 2). *Theileria ovis* was the second most prevalent species detected, with an overall exposure rate of 61.6%, where similar exposure rates were observed during both sampling periods. Two *B.*-*ovis*-positive samples (overall prevalence of 0.4%) were detected at the same farm during June (spring/early summer). During autumn, the presence of *A. bovis* was recorded in five (1.0%) samples. 

To confirm PCR–RLB and further characterize the *Anaplasma* species found, 15 out of the 22 samples sequenced were identified as *A. capra*/*A. marginale*/*A. ovis*, 3 as *A. phagocytophilum*, and *A. capra*, *A. bovis*, *A. capra*/*A. ovis*, and *A. phagocytophilum*/*A. capra*/*A. ovis* were identified.

Representative sequences of the species identified were deposited in GenBank under the accession numbers ON415279–ON415281. 

The results of binary logistic regression aiming to identify which factors influenced the presence of either *A. ovis* or *T. ovis* are presented in the Supplementary Materials (Table S1). The frequency of acaricide application (1–3 times per year) did not significantly affect the presence of either hemoparasite; thus, it was excluded from the model (data not shown). This was also the case for other variables, such as flock population, number of personnel, total pasture surface available, and altitude. The presence of *A. ovis* was influenced by two factors based on the generated model. Specifically, infestation with *R. turanicus* ticks, irrespective of the gender, was expected to result in an 8.154-fold increase in the odds in favor of *A. ovis* exposure. On the other hand, an increase in the number of *D. marginatus* males by one tick per sheep led to a 1.331-fold increase in the odds in favor of not being infected with *A. ovis.* The presence and number of the remaining tick species did not result in significant outcomes, and all such variables were excluded from the model. The generated *T.*-*ovis*-related model demonstrated that an increase in sheep infestation by *R. turanicus* females by one unit leads to an increased probability (OR: 1.191) of the presence of *T. ovis*. 

### 3.4. Detection of TBPs in R. turanicus Ticks by PCR–RLB 

Of the 169 female *R. turanicus* ticks tested, 152 (89.9%) were found to harbor at least one tick-borne microorganism belonging to the genera *Anaplasma*, *Ehrlichia*, *Babesia*, *Theileria*, or *Rickettsia*. Seventy-two ticks (42.6%) were carrying a single pathogen. The presence of multiple pathogens occurred in 47.3% of the ticks belonging to this species, with 33.7% representing dual exposure, whereas in 13.6% three pathogens were present. In Table 4, the numbers and exposure rates of single TBPs per tick species tested, without considering mixed exposure, are presented. Most commonly, the presence of unknown *Rickettsia* spp. (40.2%), followed by the presence of *A. ovis* (14.2%), *A. marginale* (24.9%), *A. phagocytophilum* (18.3%), and unknown *Anaplasma/Ehrlichia* spp. (19.5%), was observed. Comparable presence of *T. ovis* and *Babesia* spp. (*Babesia* catch-all specific signals) was observed in 24 (14.2%) and 20 (11.8%) of the ticks, respectively. In a few cases (range: 1–5/0.6–3.0%), specific signals for *Ehrlichia chaffensis*, *E. canis*, *B. major*, *T. annulata*, and unknown *Theileria* sp. were generated. 

## 4. Discussion

Exposure of livestock to ticks and, consequently, to tick-borne diseases varies between countries and regions of the world, due to differences in climatic, geographical, and environmental conditions [42,43]. This survey was conducted in Lesvos, Greece—an island with considerable sheep farming activity and favorable environmental conditions for tick propagation. It thus provides an interesting setting to study tick–pathogen–host–environment dynamics as a case scenario. As also shown in a recent review [2], such an approach provides much more than knowledge of local interest. Monitoring ticks and TBPs in the Mediterranean islands can help us to identify potential hotspots and epidemiological information that could play key roles in future control measures. The incidence of tick infestation within sheep farms and individual animals in our study was quite high, despite the regular use of acaricides by the farmers. We also recorded marked seasonal activity with regard to both intensity and tick species presence. The above suggest that the control measures applied to prevent tick infestations have not been successful, as has also been reported in the literature, and may explain the global trend of increased tick infestations in ruminants in recent decades [44,45]. The higher tick infestations seen during spring/early summer are probably due to ticks’ biology and environmental conditions (e.g., optimal ambient temperature)—especially today, when the effects of climate change are more obvious [3]. Another factor that makes the above situation even harder to tackle is that spring is actually the peak of animals’ lactation period. During this time, treatments in dairy flocks are limited, since the use of several acaricides is restricted by EU legislation (with regard to the presence of harmful residues in the milk).

Overall, nine different tick species belonging to the genera *Rhipicephalus*, *Hyalomma*, *Haemaphysalis*, and *Dermacentor* were identified. All of the above were previously reported in other studies performed on both the mainland and islands in Greece [15,17,18,19,46]. All of the ticks that we found were adults; however, this did not come as a surprise, since the sampling period started in May, and immature stages are expected to be present earlier in the season [3]. The predominance of *R. turanicus* in terms of both numbers and distribution during the spring/early summer months was also documented in studies performed in other countries around the Mediterranean Basin and neighboring sites, such as the Marmara region of Turkey and Cyprus [47,48,49,50,51]. Apart from Mediterranean countries, the distribution of this species extends to both Asia and Africa [52,53,54], and is known for the wide range of hosts—including humans—that it is capable of infesting [14,55,56]. However, a recent study challenged this hypothesis, supporting a taxonomic separation of *R. turanicus* ticks with an origin from the Afrotropics (suggesting the presence of a separate species named *R. afranicus* n. sp. therein), thus restricting the *R. turanicus* sub-lineage’s distribution to the Palearctic [56]. *Dermacentor* *marginatus* and *H. parva* were mostly present during the autumn collection period, with the former demonstrating considerable infestation intensities when present and a wide distribution on the island compared to studies performed on the mainland during autumn or winter months, where a sparser distribution was demonstrated [15,17,19]. This species was found at altitudes ranging from 4 to 574 (the highest sampling altitude of the present study) meters above sea level (asl). It should be mentioned that previous studies additionally or exclusively focused on higher altitudes [18,19]. Interestingly, *H. parva* (syn. *H. otophila*)—the second most common species collected during autumn—was mainly found along the northern/northeastern part of the island, where deciduous oak woodlands prevail. It was found at altitudes ranging between 25 and 570 meters asl, in contrast with a previous finding from Greece stating its presence exclusively at high altitudes [19]. *Haemaphysalis parva*, a three-host tick, seems to have a wide geographic distribution, being mainly found in Middle Eastern countries, and infesting domestic and wild animals, birds, and even humans [55,57,58,59,60,61]. Previous studies from Greece, Italy, Croatia, and Romania point to a rather sparse distribution of this species when collected from ruminants or by dragging [15,17,19,62,63,64]. However, a recent study demonstrated frequent infestation of temporary kennel dogs in Northern and Central Greece [21]. The scarce infestation of sheep with *Hyalomma* ticks confirms previous observations from this country [15,18,19].

Tick-borne-pathogen-related epidemiological data on small ruminants in Greece remain limited. Our data revealed high exposure rates (84.9%) of sheep, with the majority showing a concurrent presence of different TBPs (58.2% prevalence). The highest frequency of co-exposure was observed between *A. ovis* and *T. ovis*, which were also the most frequently encountered TBPs, demonstrating RLB-based single exposure prevalence of 77.2% and 61.6%, respectively. *Anaplasma* ovis is widely distributed in the Mediterranean Basin, causing ovine anaplasmosis, and seems to be more host-specific, affecting ovine and caprine erythrocytes [6,65,66]. High prevalence of *A. ovis*, either as a single exposure or combined with *T. ovis*, was previously reported in several countries around but not limited to the Mediterranean Basin [2,66,67,68,69,70,71,72]. *Anaplasma ovis* infections are considered to induce only mild clinical symptoms and, thus, are of minor economic importance; however, several cases have reported clinical signs in sheep such as severe anemia, extreme weakness, anorexia, and weight loss [73,74,75,76,77], but disease mainly occurs under poor flock management conditions [65,75].

In Greece, *A. ovis* infection has so far been demonstrated within the framework of cross-sectional studies in cattle and one goat herd (by serology and blood smears, respectively), but only in northern parts of the country [22,78]. The majority (88.4%) of the enrolled flocks in our study consisted of animals belonging to the local Lesvos breed. Even though our study was performed on randomly selected, apparently healthy adult (>12 months) animals, the high *A. ovis* exposure rates raise questions about possible production losses, as outbreaks of anaplasmosis due to this pathogen were previously described in Greece [79]. However, it should be noted that farmer questionnaires and feedback from local vets noted a tolerance of the local breed against TBPs. This is not unexpected, since previous research demonstrated that sheep breed might play an important role in tolerance to TBPs [80]. After experimental infection with *A. ovis* in different sheep breeds—both local and imported—all of them developed the disease. However, symptoms, along with hematological and clinical parameters, varied depending on the breed [80]. Apart from small ruminants’ pathogenicity to *A. ovis*, a few studies illustrated susceptibility of humans or human cell lines to infection with this TBP, with a case of a woman from Cyprus presenting clinical symptoms due to this TBP [81,82,83]. 

Notably, sequencing results of *Anaplasma*/*Ehrlichia* spp. samples not defined by RLB in sheep and in *R. turanicus* ticks showed the presence of *A. phagocytophilum*—the etiological agent of tick-borne fever in sheep and human granulocytic anaplasmosis, which is an emerging tick-borne disease of humans. In Europe, *A. phagocytophilum* is mainly transmitted by the tick species *Ixodes ricinus* [6,84]. Young animals are mostly affected by *A. phagocytophilum* infection, and clinical signs include high fever, anorexia, sudden drops in milk yield, abortion in ewes, and reduced fertility in rams [85,86]. Clinical cases due to this pathogen in both humans (Crete and Central Greece) [87,88] and sheep (in Northern Greece) [24], as well as infection of both *I. ricinus* and *R. bursa* ticks, were previously demonstrated for Greece [89]. The cosmopolitan nature of *A. phagocytophilum* and its detection in species other than those belonging to the *I. persuculatus* complex might indicate their implication in the transmission of this TBP [90]. On top of the above findings, our sequencing results revealed the presence of *A. capra*, and this is, to the best of our knowledge, the first report of this pathogen in Greece. *Anaplasma capra* was recently (2012) recognized as a separate species after being isolated from goats in China, followed by several other reports from various other countries, including European countries. *Anaplasma capra* has been found to infect humans, goats, sheep, cattle, dogs, and wild animals [11,12,91,92,93].

Lastly, the presence of *A. bovis* was also documented in the present study, which also seems to occasionally occur in small ruminants [94]. To our knowledge, this is the first molecular confirmation of this species in Greece. Overall, the results of the present study suggest that further research to investigate the molecular characterization, dissemination, vectors, and clinical and economic impact of different *Anaplasma* species is essential—especially since all species isolates can have adverse effects on human health as well. 

The generated binary logistic regression model supports a role of *R. turanicus* ticks in *A. ovis* transmission, as animals infested with this tick species were 8.154 times more likely to be infected with this TBP. In addition, *A. ovis* exposure rates were significantly higher during spring/early summer compared to the autumn collection period (93.8% vs. 66.7%), with the former corresponding to the period of nearly absolute *R. turanicus* predominance, whereas *R. bursa* ticks—also a suspected *A. ovis* vector—demonstrated a low exposure intensity when present, and a sparser distribution. The above findings support an important role of this tick species in *A. ovis* transmission on the island. However, additional tick species belonging to the genera *Rhipicephalus* and *Haemaphysalis*, as well as *D. marginatus*, are considered to be possible vectors of *A. ovis* in the Mediterranean Basin, thus generating a tangled situation [57,70,71,95,96,97]. A common characteristic of *Anaplasma* spp. infections is the development of persistent infections in the vertebrate host which, in turn, allows them to serve as reservoirs of infection [98]. *Anaplasma* spp. seem to be transmitted horizontally by ixodid ticks, while transovarial transmission does not appear to occur [99]. Transstadial transmission takes place from stage to stage; therefore, every tick generation must obtain infection by feeding on reservoir hosts, e.g., as observed for *A. phagocytophilum*, which has been detected in the blood of a wide range of mammals. For ruminant-host-specific *Anaplasma* spp., such as *A. marginale*, intrastadial transmission seems to be influenced by male ticks (intermittent feeders), as previously demonstrated for *D. andersoni*, which can both acquire infection and, during a second feeding, transmit the pathogen [100]. Therefore, a similar transmission mechanism could also be of relevance for *R. turanicus*, even though our model did not point to a gender-related transmission pattern. In addition, the fact that some tick species can parasitize sheep both as adults and at immature stages at different points of the year—e.g., as observed for *R. bursa* and *H. punctata* [36,101]—does not rule out the possibility of transstadial transmission. The negative association between the increasing number of *D. marginatus* male ticks and *A. ovis* exposure, as revealed by our model, might point to a competitive exclusion mechanism, which we are unable to explain sufficiently. However, all animals infested with *H. parva* only during the month of high activity of autumn ticks (October) were exposed to *A. ovis*, which was a significant difference (data not shown). On the other hand, concurrent infestation of animals with *H. parva* and *D. marginatus* or *D. marginatus* only did not result in a significant difference between the number of *A.*-*ovis*-exposed and non-exposed animals. In any case, all of the above remain to be addressed, since experimental infection models with the above tick species and their respective TBPs are lacking in the literature. 

Ovine babesiosis due to *B. ovis* represents an important impediment to meat and milk production, because infected small ruminants exhibit high parasitemia and mortality [96,102]. Outbreaks due to this protozoan pathogen have been reported in many countries around the Mediterranean—such as Turkey, Israel, Italy, and Spain—and severe clinical or even fatal cases occur frequently [102,103,104,105,106]. A previous serological study in Northern Greece demonstrated that *B. ovis* and *T. ovis* (52.1% and 25.5% prevalence for sheep, respectively) were widespread in sheep [22]. A more recent PCR-based approach in Central and northwestern Greece revealed considerable prevalence rates (15.1%) of *B. ovis* in small ruminants [23]. However, the results of the present study demonstrate low *B. ovis* prevalence rates (0.4%), in accordance with the low numbers of *R. bursa*—the vector of this pathogen—found on the island [107,108,109]. 

## 5. Conclusions

The data presented in this study reveal a high burden of ticks and TBPs in sheep in Lesvos, suggesting that the island may serve as a hotspot, supporting their further expansion due to its geographic location. Nine tick species were found parasitizing animals. However, the predominance of *R. turanicus*—especially during the spring/summer months—was quite evident. The main TBP genera detected were *Anaplasma* and *Theileria*, which have a great importance in both veterinary and human health. Control measures of both ticks and TBPs seem to be failing, as shown by their high endemicity—possibly because they focus on controlling tick infestations using classic acaricides, without considering environmental conditions, tick ecology, or vector–pathogen interactions. This, in addition to the animals’ health/welfare and economic impact, may affect public health. Therefore, there is a specific need for better design of monitoring and control programs using a more holistic One Health approach.

## Figures and Tables

**Table 1:** (**a**) Distribution (rate, and total number in brackets) of tick species collected per month and total prevalence for the whole collection period are given. (**b**) Infestation rates at the animal or farm level stratified by month, collection period, or the whole study are additionally provided.

(a)					
Species/Month	May	June	September	October	Whole Period
	Spring/Summer		Autumn		
*Rhipicephalus turanicus*	95.2%	84.5%	-	-	61.3%
	(*n* = 589)	(*n* = 457)			(*n* = 1046)
*Rhipicephalus bursa*	0.8%	10.0%	4.4%	-	3.6%
	(*n* = 5)	(*n* = 54)	(*n* = 2)		(*n* = 61)
*Rhipicephalus sanguineus* sensu lato	4.0%	0.2%	-	-	1.5%
	(*n* = 25)	(*n* = 1)			(*n* = 26)
*Hyalomma marginatum*	-	3.9%	-	-	1.2%
		(*n* = 21)			(*n* = 21)
*Hyalomma excavatum*	-	1.5%	-	-	0.5%
		(*n* = 8)			(*n* = 8)
*Dermacentor marginatus*	-	-	57.8%	48.0%	15.6%
			(*n* = 26)	(*n* = 241)	(*n* = 267)
*Haemaphysalis parva*	-	-	37.8%	44.2%	14.0%
			(*n* = 17)	(*n* = 222)	(*n* = 239)
*Haemaphysalis punctata*	-	-	-	7.6%	2.2%
				(*n* = 38)	(*n* = 38)
*Haemaphysalis sulcata*	-	-	-	0.2%	0.1%
				(*n* = 1)	(*n* = 1)
Total	*n* = 619	*n* = 541	*n* = 45	*n* = 502	*n* = 1707
**(b)**					
**Infestation rate (sheep)**	96.7%	89.5%	16.3%	48.7%	55.4%
	(*n* = 90)	(*n* = 105)	(*n* = 160)	(*n* = 150)	(*n* = 505)
	92.8%		31.9%		
	(*n* = 195)		(*n* = 310)		
**Infestation rate (farms)**	100%	100%	37.5%	73.3%	72.3%
	(*n* = 18)	(*n* = 21)	(*n* = 32)	(*n* = 30)	(*n* = 101)
	100%		54.8%		
	(*n* = 39)		(*n* = 62)		

**Table 2:** Presence of single pathogens and their respective rates in sheep (multiple presence not considered), stratified based on species and collection period. Different superscripts (a and b) indicate significant differences at the *p* < 0.001 level (abbreviations: CI, confidence interval).

	Spring/Early Summer	Autumn	Whole Period
Tick-Borne Pathogens	Number of Positive Samples (Exposure Rate; 95% CI)		
*Anaplasma ovis*	183 ^a^ (93.8%)	207 ^b^ (66.7%)	390 (77.2%; 73.4–80.8.)
*Theileria ovis*	125 ^a^ (64.1%)	186 ^a^ (60.0%)	311 (61.6%; 57.2–65.7)
*Babesia ovis*	2 ^a^ (1.0%)	0 ^a^ (0%)	2 (0.4%; <0.01–1.5)
*Anaplasma bovis*	0 ^a^ (0%)	5 ^a^ (1.6%)	5 (1.0%; 0.4–2.4)
*Anaplasma/Ehrlichia* spp. unknown	2 ^a^ (1.0%)	8 ^a^ (2.6%)	10 (2.0%; 1.0–3.7)
*Babesia/Theileria* spp. unknown	6 (3.1%)	3 (1.0%)	9 (1.8%; 0.9–3.4)
Number of samples tested	*n* = 195	*n* = 310	*n* = 505

**Table 3:** Prevalence of single and mixed infections (including 95% CIs) detected in sheep during the study period (abbreviations: CI, confidence interval).

	Number of Cases	Prevalence (95% CI)
**No infection**	76	15.1% (12.2–18.4)
**Single infections**	135	26.7% (23.1–30.8)
*Anaplasma ovis*	108	21.4% (18.0–25.2)
*Theileria ovis*	26	5.1% (3.5–7.5)
Unknown *Babesia/Theileria* spp.	1	0.2% (<0.01–1.2)
**Double infections**	290	57.4% (53.3–61.9)
*Anaplasma ovis + Theileria ovis*	270	53.4% (51.1–59.7)
*Unknown Anaplasma/Ehrlichia* spp./*Theileria ovis*	10	2.0% (1.0–3.7)
*Anaplasma ovis + Babesia ovis*	2	0.4% (0.01–1.5)
*Anaplasma ovis +* unknown *Babesia/Theileria*	6	1.2% (0.5–2.6)
*Anaplasma bovis + Theileria ovis*	1	0.2% (<0.01–1.2)
*Anaplasma bovis +* unknown *Babesia* spp.	1	0.2% (<0.01–1.2)
**Triple infections**	4	0.8% (0.2–2.1)
*Anaplasma ovis + Anaplasma bovis + Theileria ovis*	3	0.6% (0.1–18)
*Anaplasma ovis + Theileria ovis +* unknown *Babesia* spp.	1	0.2% (<0.01–1.2)
**Total**	505	100.0

**Table 4:** Numbers and exposure rates (including 95% CIs) of single tick-borne pathogens detected in female *Rhipicephalus turanicus* ticks collected during the study (abbreviations: CI, confidence interval).

	*R. turanicus*
Number of ticks tested (females/males)	*n* = 169 (169/0)
**Tick-borne pathogens**	**Number of positive samples (exposure rate; 95% CI)**
*Anaplasma ovis*	24 (14.2%; 9.7–20.0)
*Anaplasma phagocytophilum*	31 (18.3%; 13.2–24.9)
*Anaplasma marginale*	42 (24.9%; 18.9–31.9)
*Ehrlichia chaffensis*	5 (3.0%; 1.1–6.9)
*Ehrlichia canis*	1 (0.6%; <0.01–3.6)
*Anaplasma/Ehrlichia* spp. unknown	33 (19.5%; 13.2–24.9)
*Rickettsia* spp. unknown	68 (40.2%; 33.1–47.8)
*Babesia major*	4 (2.4%; 0.7–6.1)
*Theileria ovis*	24 (14.2%; 9.7–20.3)
*Theileria annulata*	3 (1.8%; 0.4–5.3)
*Babesia/Theileria spp.* unknown	-
*Babesia* spp. unknown	20 (11.8%; 7.7–17.6)
*Theileria* spp. unknown	5 (3.0%; 1.1–6.9)

## Supplementary Materials

The following supporting information can be downloaded at: www.mdpi.com/article/10.3390/microorganisms10081551/s1, Table S1: Binary logistic regression models related to *Anaplasma ovis* and *Theileria ovis* infection presence in sheep.
