# Supplementary material for: The Scenario of Ticks and Tick-Borne Pathogens of Sheep on a Mediterranean Island
Source: Microorganisms. 2022 Jul 31;10(8):1551. doi: 10.3390/microorganisms10081551 (PMC9412349; doi:10.3390/microorganisms10081551)
Supplement: Supplementary file 1 [file microorganisms-10-01551-s001.zip › microorganisms-1803348-supplementary.pdf]

**Table S1.** Binary logistic regression models related to *Anaplasma ovis* and *Theileria ovis* infection presence in sheep

| ***A. ovis* model** | | | | | | | **95% CI** | |
|---|---|---|---|---|---|---|---|---|
| **Variable** | **B** | **SE** | **Wald** | **Df** | **p** | **OR** | **Lower** | **Upper** |
| *Rhipicephalus turanicus* presence: yes | 2.099 | 0.445 | 22.280 | 1 | <0.001 | 8.154 | 3.412 | 19.490 |
| No of *Dermacentor marginatus* males | -0.286 | 0.141 | 4.150 | 1 | 0.042 | 0.751 | 0.570 | 0.989 |
| Constant | 1.080 | 0.155 | 48.462 | 1 | <0.001 | 2.943 | | |
| $R^2$ (Nagelkerke): 17.9%<br>Omnibus Tests of Model Coefficients: $\chi^2$ (2) = 44.778, p< 0.001<br>H-L test: $\chi^2$ (1) = 0.169, p = 0.681 | | | | | | | | |
| **T. ovis model** | | | | | | | | |
| No of *R. turanicus* females | 0.175 | 0.59 | 8.691 | 1 | 0.003 | 1.191 | 1.060 | 1.337 |
| Constant | 0.322 | 0.118 | 7.473 | 1 | 0.006 | 1.379 | | |
| $R^2$ (Nagelkerke): 4.2%<br>Omnibus Tests of Model Coefficients: $\chi^2$ (1) = 12.150, p< 0.001<br>H-L test: $\chi^2$ (2) = 1.468, p = 0.480 | | | | | | | | |
